# Laser Polishing of Vertically Oriented FDM-PLA Components: Influence of Laser Power and Polishing Speed on Surface Topography and Mechanical Response

**DOI:** 10.3390/polym17233096

**Published:** 2025-11-21

**Authors:** Gabriel Stolárik, Radoslav Vandžura, Róbert Ropovík, Damián Peti, Matúš Geľatko

**Affiliations:** Faculty of Manufacturing Technologies with a Seat in Prešov, Technical University of Kosice, Štúrova St. 31, 080 01 Prešov, Slovakia; gabriel.stolarik@tuke.sk (G.S.); damian.peti@tuke.sk (D.P.);

**Keywords:** post-processing, laser polishing, scanning speed, laser power, roughness, strength

## Abstract

Laser polishing represents a promising post-processing method for FDM-printed parts, enabling targeted manipulation of their surface topography and the associated functional properties. Despite extensive research in this field, no experimental investigation has yet addressed the configuration with vertical build orientation, which is critical in terms of interlayer stress transfer. Therefore, this study focuses on CO_2_ laser polishing of vertically printed PLA specimens at power levels of 10 and 12 W and polishing speeds of *v_f_* = 200, 400, and 600 mm·s^−1^, with a constant transverse displacement of 0.1 mm. The results showed that both laser power and polishing speed have a significant influence on the surface topography and mechanical properties of the samples. The lowest roughness values, *R_a_* = 10.24 ± 0.14 µm at 10 W and *R_a_* = 12.20 ± 0.43 µm at 12 W, were achieved at the highest polishing speed of *v_f_* = 600 mm·s^−1^; however, these still exceeded the roughness of the reference unpolished sample (*R_a_* = 9.02 ± 0.21 µm). The increase in roughness was attributed to the formation of relief structures caused by insufficient overlap of laser passes, orthogonal to the printing-induced surface orientation. These reliefs further acted as local stress concentrators, resulting in a decrease in mechanical performance of up to 9% in comparison with the reference specimen. At 10 W, the surface profile was dominated by *R_v_* > *R_p_*, similar to the reference sample, whereas at 12 W, the opposite trend (*R_p_* > *R_v_*) was observed—attributed to more intense subsurface melting and valley filling by the melt pool. Therefore, the findings clearly confirm that the critical factor is the energy distribution and transverse displacement of the laser polishing paths, where improper parameter settings may visually improve the surface while structurally weakening it. These findings highlight the need for further research, focused on the precise optimization of the polishing trajectory and the energy distribution for vertically oriented samples.

## 1. Introduction

The FDM (Fused Deposition Modeling) process is an additive manufacturing (AM) technique based on the reproduction of a 3D CAD model. In this method, a component of the desired geometry is fabricated by sequential depositing of individual material layers. During the printing, the feedstock material is extruded through a high-temperature nozzle, which melts it and enables the bonding with the previously solidified layer [1]. Considering the advantage of relatively low extrusion temperatures (typically below 250 °C), which allows operation under non-specialized environmental and machine conditions [2], environmentally friendly thermoplastic materials such as polylactic acid (PLA) and acrylonitrile-butadiene-styrene (ABS) are commonly employed [3]. Due to their availability and ecological character, these materials are primarily used for prototyping applications. However, when the printed part is intended to serve as a functional component within an industrial assembly (e.g., automotive, aerospace, medical sector), ensuring its long-term durability becomes essential. Multiple studies have demonstrated that the durability of FDM parts can be improved from several perspectives.

The first option is reinforcing the filament with fibers, forming a composite material [4,5]. It has been shown that PLA composites reinforced with carbon, glass, or metal fibers exhibit increased strength and improved mechanical performance [6,7]. In addition to the material selection, the mechanical strength and stiffness of the printed part are significantly influenced by printing parameters. The fundamental parameters include the layer height [8], which affects the volumetric density of the part and is primarily determined by the nozzle diameter. In general, smaller layer heights enable fabrication of dimensionally accurate parts with a better surface quality, at the expense of longer production times [9], whereas larger layers are preferred for faster prototyping [10]. The printing time is strongly governed by the deposition feed rate, which also influences residual stresses and deformation of the part [11]. The feed rate is closely related to the filament extrusion rate, and any mismatch between these two may result in the poor interlayer bonding and delamination [12]. The extrusion rate is dependent on the nozzle temperature [13], where higher temperatures improve dimensional accuracy [14]. Other important factors include the raster angle and build orientation, which affect not only tensile behavior, but also fracture characteristics [15]. Several studies have shown that as raster angle increases (0–90°), flexural and tensile strength decrease, which has been attributed to the reduction in effective fiber length for stress transfer [15,16,17]. The dominant influence of raster angle on tensile strength was also confirmed in [18], where results showed that stress transfer is the most efficient at 0–45° due to layer orientation parallel to the tensile loading axis, whereas the worst performance occurred at 45–90° due to weak interlayer bonding, often leading to the delamination. Similar behavior was observed during the changing of build orientation to the XZ or YZ planes, where vertical (*Z*-axis) printing reduced Young’s modulus in standard ASTM D638 tensile specimens, but increased maximum strain capacity in more complex geometries [19]. Consequently, consensus exists in the literature, where the highest tensile strength is achieved when the raster angle and print orientation are aligned with the loading direction [1,17,20,21].

Beyond material and printing parameters, mechanical properties can also be enhanced through the post-processing [22]. Laser polishing has been identified as a highly effective post-processing method for FDM parts [23] using CO_2_ [24], diode [25], femtosecond [26] and fiber [27,28] lasers. It is regarded as an attractive technique for improving mechanical properties via surface layer remelting, performed without a tool wear, making it environmentally sustainable [24]. Remelting the surface layers enhances interlayer bonding, thus reducing delamination and simultaneously lowering surface roughness [29]. When the laser polishing is considered during process planning, it becomes possible to utilize faster printing parameters (larger layer height, higher polishing speed), thereby reducing the FDM processing time and improving overall time efficiency [30].

The reviewed studies clearly demonstrate the availability of multiple strategies for improving the performance of FDM parts. One of the most attractive approaches is combining accelerated printing parameters with post-processing, as this allows simultaneous reduction in build time, improvement of mechanical strength, and reduction in surface roughness via laser polishing. Previous studies focused on laser polishing of PLA [24,29,30,31,32] have reported favorable results, predominantly on horizontally printed specimens. However, real industrial applications often involve complex geometries printed across multiple axes, including the vertical direction. The vertical layer orientation is well known for significant reduction in mechanical strength, particularly tensile strength, due to weaker interlayer adhesion. It also produces a pronounced stair-stepping effect on side surfaces, which negatively impacts surface quality. It has been shown that printing at an angle of 60° can improve the surface morphology through enhanced structural consolidation [23]. Nevertheless, no study to date has investigated the effectiveness of laser polishing on vertically printed samples.

Therefore, the aim of this study is to analyze and optimize the laser post-processing of FDM parts with vertical layer orientation. During the experiment, the laser beam was directed orthogonally to the print direction, intentionally supporting the surface remelting and the flow of material into valleys. Laser powers of *P* = 10 and 12 W were tested in a combination with polishing speeds *v_f_* = 200, 400, and 600 mm·s^−1^.

## 2. Materials and Methods

### 2.1. Material and 3D Printing Method

In this experiment, PLA Elegoo PLA MATTE filament with a diameter of 1.75 mm was used as the printing material, without any fillers or additional modifiers. This approach enables an extraordinary evaluation of the effect of laser polishing on the mechanical properties of the specimens, as the presence of reinforcing agents in composite materials could introduce distortions arising from interactions between the matrix and filler. By employing a pure material, the influence of secondary factors is eliminated, ensuring a clear and unambiguous assessment of the polishing process. Prior to printing, the filament was dried at 50 °C to a moisture level of 8%, minimizing water absorption and preventing printing defects. The filament color was gray, selected due to its advantageous absorption properties under CO_2_ laser irradiation [31]. It is important to say that all samples were printed from a single roll of filament, which minimizes the impact of different material properties on the resulting mechanical properties of the specimens.

**Figure 1:**
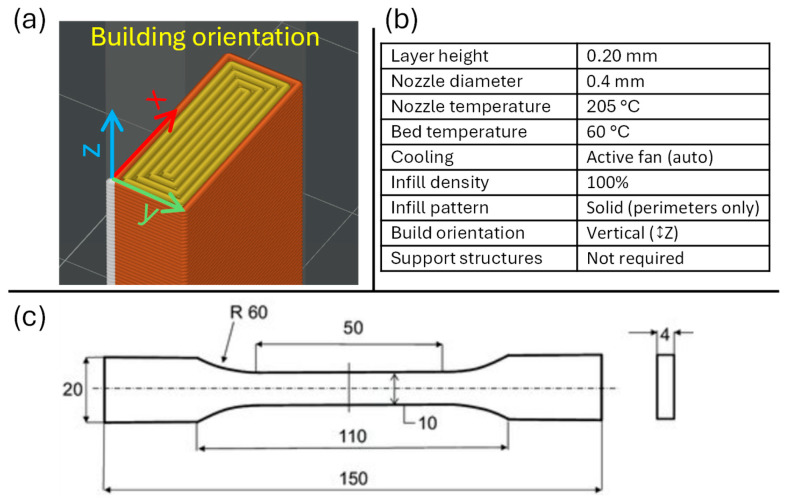
(**a**) Printing orientation process, (**b**) 3D printing parameters, (**c**) sample dimensions according to ISO 527-2 1B.

The specimens were printed using a Bambu Lab X1 Carbon (X1C) system, which features high positioning accuracy and a closed printing chamber, thereby minimizing the influence of environmental fluctuations on process stability. The specimens were prepared in Bambu Studio slicer, based on a 3D model designed in Autodesk Inventor 2025. The printing parameters are shown in Figure 1b. To ensure reliable bed adhesion, a “Cool Plate” was used in combination with a brim, which prevented deformation and detachment during the printing process. The samples were printed in batches of 10 in immediate succession to minimize the influence of the printer’s internal environment, with the test specimens oriented vertically on the build plate, in a way that the layer deposition occurred in the horizontal X–Y plane, while the model grew along the *Z*-axis (Figure 1a).

The geometry and dimensions of the tensile specimens were designed according to the ISO 527-2:2012, type 1B standard [33] (Figure 1c), which is intended for plastics produced by additive manufacturing or machining. This specimen type features a nominal cross-section of 10 × 4 mm and a gauge length of 50 mm, enabling reliable determination of tensile behavior. ISO 527-2 [33] specifies the methodology for determination of tensile strength, elastic modulus, and elongation of polymeric materials, ensuring the comparability of results across different thermoplastics.

### 2.2. Laser Polishing

**Figure 2:**
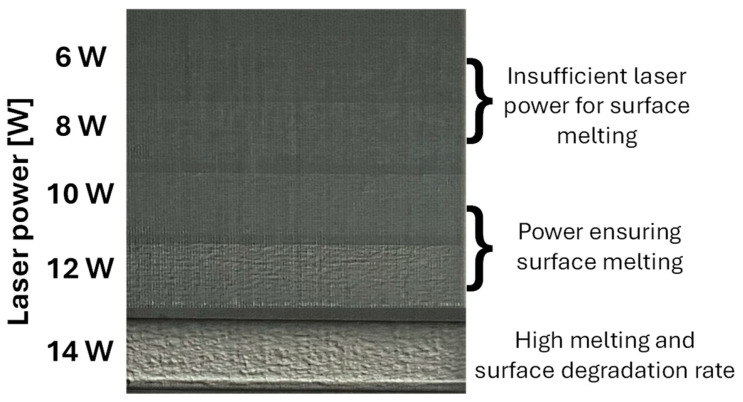
Primary experiment for evaluating laser power “*P*” at a polishing speed *v_f_* = 200 mm·s^−1^.

Laser polishing was carried out using a Thunder Laser CO_2_ system, model Nova 51, equipped with air-assisted flow through a nozzle. The machine features an integrated CO_2_ laser tube with a maximum power of 100 W, a working area of 1300 × 900 × 230 mm, and a maximum speed of 1000 mm·s^−1^. The engraving program was generated using the ThunderLaser (V8 2021) proprietary software, which is directly compatible with the system. The variable laser parameters were set to power levels of *P* = 10 W and 12 W (corresponding to 10% and 12% of the machine’s maximum output) and three polishing speeds of *v_f_* = 200, 400, and 600 mm·s^−1^. The laser power was carefully selected based on preliminary tests. These preliminary experiments included a primary experiment in which power levels of *P* = 6, 8, 10, 12, and 14 W were evaluated at a polishing speed of *v_f_* = 200 mm·s^−1^ (see Figure 2). The results showed that powers below 10 W did not produce sufficient surface melting or modification, while powers above 12 W led to surface degradation. Therefore, for the purposes of this study, only 10 W and 12 W were selected, combined with the variable polishing speeds described above. These speed–power combinations were intentionally selected to provide sufficient experimental variability for observing the differences between complete surface melting and mere superficial polishing.

**Figure 3:**
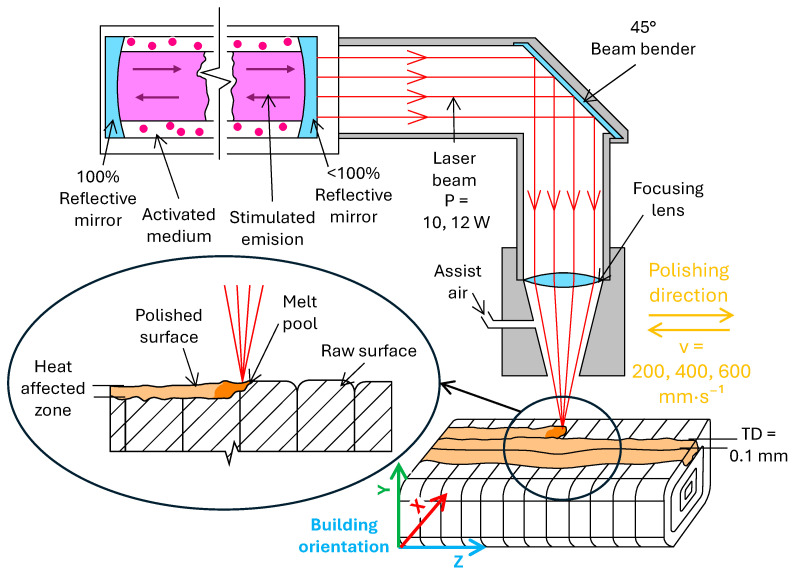
Laser polishing process of vertically printed PLA materials.

The fixed parameters included a standoff distance of 5 mm and a transverse displacement (distance between adjacent passes) of *TD* = 0.1 mm which was defined as optimal based on studies [24,30,34]. Polishing was performed on both, the upper and lower surfaces of the specimens, with the laser polishing path oriented perpendicular to the printing direction (see Figure 3). Each laser parameter configuration was applied to five individual specimens, ensuring repeatability of the experiment and enabling the acquisition of data suitable for subsequent statistical analysis.

### 2.3. Measurement Methods

Surface characterization—Optical imaging, 3D surface reconstruction, and roughness measurements were performed using a KEYENCE VHX-7000 digital microscope. Surface roughness was evaluated based on profile roughness parameters following application of S-filter and F-filter processing in accordance with the ISO 21920–2 [35]. The arithmetical mean height (*R_a_*)—widely used in both scientific and industrial contexts—was selected as the primary evaluated parameter. However, since *R_a_* individually may provide limited and potentially misleading insight into the surface state, the maximum height average (*R_z_*) was additionally assessed, representing the mean difference between the five highest peaks and five deepest valleys along the evaluation length. For qualitative interpretation of surface morphology, the separate peak (*R_p_*) and valley (*R_v_*), height parameters were also included. Roughness was measured on every specimen, and the resulting values were statistically and graphically processed, reported as mean ± standard deviation.

**Figure 4:**
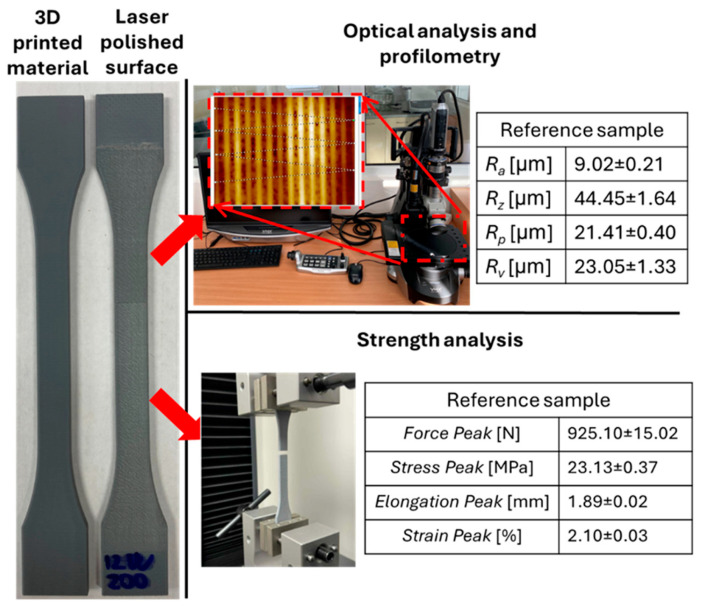
Methods for characterizing and measuring samples with input parameters of the raw surface of reference samples after printing process.

Mechanical testing—Tensile tests were performed using a Testometric X350 universal testing machine (Testometric Co Ltd., Rochdale, UK), equipped with a high-resolution electronic force measurement system, a 5000 N load cell, and fully digital control adaptable to the specified test parameters. All tests were conducted under standard laboratory conditions at 22 °C and relative humidity of 55 ± 5%. Following ISO 527 [33] guidelines, a test speed of 20 mm·min^−1^ was selected—within the recommended 5–50 mm·min^−1^ range for thermoplastics such as PLA. The tensile data were recorded in real time using the Testometric WinTest Analysis software (version 6.0.17.1). Figure 4 illustrates the measurement process and presents the referential (unpolished) specimen’s surface roughness and tensile strength values.

## 3. Results and Discussion

### 3.1. Optical Analysis and 3D Reproduction of Surfaces

**Figure 5:**
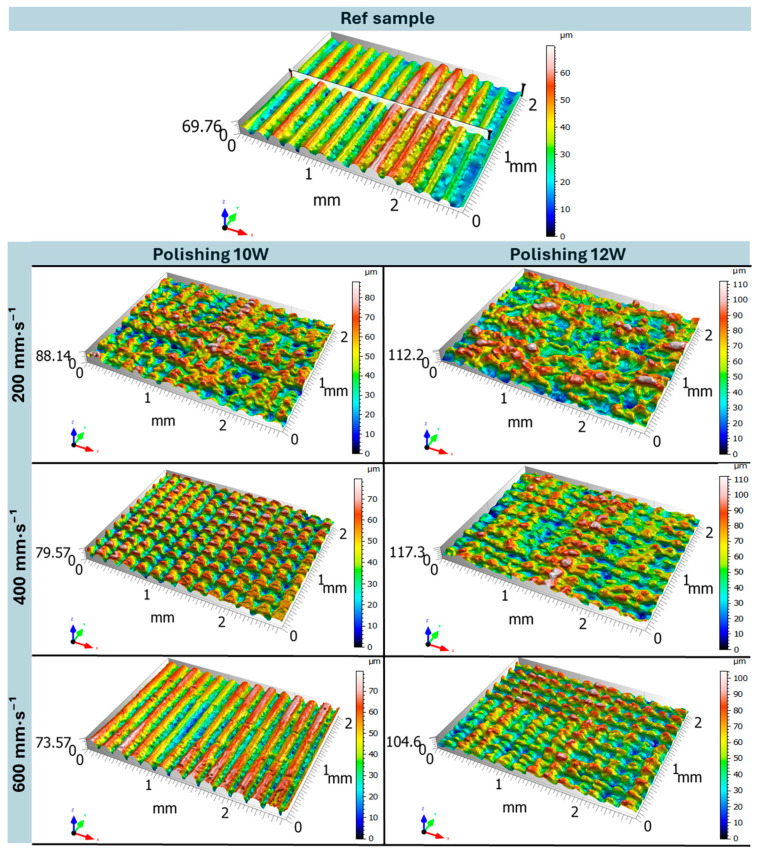
Optical 3D reconstruction analysis of the reference sample and samples after laser polishing with different polishing parameters.

Figure 5 shows the optical 3D surface reconstruction of the samples. Surface topography was scanned prior to destructive testing—immediately after the additive manufacturing process (reference specimen)—and subsequently after laser polishing. The scan of the reference sample clearly reveals a pronounced periodic surface structure corresponding to the individual layers formed during the FDM printing process. This wavy topography is a direct consequence of the layer-by-layer deposition of extruded thermoplastic (PLA), where the height and spacing of the waves correspond to the programmed layer thickness during material deposition [36]. Upon closer observation, the peak regions exhibit a smoother and more compact morphology, free from notable defects or cracks, whereas the valley regions appear irregular and display a more chaotic and porous microstructure. This behavior is associated with variations in interlayer bonding strength, which depends on local temperature, cooling rate, and extrusion parameters (primarily extrusion speed) [37]. Due to the dynamic nature of the extrusion process, local over-extrusion [38] may occur in certain regions, where the volume of molten material slightly exceeds the feed rate of the nozzle. As a result, the filament spreads laterally, forming characteristic wavy edges. Conversely, in regions affected by under-extrusion [38], the deposited material is insufficient, leading to deeper valleys and reduced surface homogeneity. Profilometric analysis of the 2D cross-section shows that the depth of the valleys is not constant. This phenomenon can be attributed to fluctuations in the ratio between nozzle printing speed and material extrusion rate. In regions with greater valley depth, a higher nozzle feed rate or lower local extrusion probably occurred, whereas shallower valleys indicate more stable extrusion and improved interlayer fusion.

The surface topography analysis after laser polishing revealed distinct morphological variations depending on the applied laser power (*P* = 10 W and 12 W) and polishing speed (*v_f_* = 200, 400, and 600 mm·s^−1^), relative to the untreated reference sample. Considering that the polishing process was performed perpendicular to the build orientation, the original layer-induced linear surface pattern was disrupted by the transverse laser passes during surface polishing [23]. In all tested cases (*v_f_* = 200, 400, and 600 mm·s^−1^), the height map exhibited a greater amplitude variation at 12 W compared to 10 W, which can be attributed to the more intense melt pool formation at higher laser power—resulting from the increased energy input. Consequently, the intensity of the melt pool directly influenced both the surface quality and the topographic structure. At the lowest polishing speed of *v_f_* = 200 mm·s^−1^, 10 W caused significant surface damage, with excessive melting of the original peaks (red regions) extending deep into the lower valley zones (blue regions). It is important to note that these valleys originate from the porous structure formed during printing (as described previously). When the laser interacts with this porous microstructure, it melts and consolidates it more rapidly, which may further explain the increased depth variation compared to the untreated sample. The resulting topography resembles a partially repeating pattern of separated peaks, with localized melting disrupting the original FDM-printed periodicity. In contrast, at 12 W and the same polishing speed, the surface morphology became highly stochastic and non-uniform, characterized by aggressive melting and uncontrolled merging of adjacent peaks—resulting in an anisotropic and microstructurally unstable surface. As the polishing speed increased to *v_f_* = 400 mm·s^−1^, the polishing effect was noticeably reduced due to the shorter laser–material interaction time. At 10 W, only slight melting and rounding of peak tips occurred, without reaching the valley regions, resulting in a regular and periodically repeating surface profile with clearly defined peak boundaries. Reversibly, at 12 W, melt pool once again penetrated into the lower layers, producing a surface somewhat comparable to 10 W at *v_f_* = 200 mm·s^−1^, though visually more homogeneous and with reduced surface amplitude. At *v_f_* = 600 mm·s^−1^, the surface morphology at 10 W remained largely dominated by rounded peaks originating from the original FDM process, with only sporadic and shallow melt tracks occurring randomly across the surface. Under the same polishing speed but at 12 W, the peak activity remained pronounced, but the peak geometry became sharper and more asymmetric. This behavior is attributed to the fact that higher laser power increases the size of the heat-affected zone, leading to partial melting of the lateral peak walls, causing material flow and peak skewing.

Interestingly, increasing the polishing speed did not lead to noticeable changes in the height map. This indicates that in both cases, the laser power was sufficiently high to melt the arc-shaped peaks down to their lower regions, where the porous structure was simultaneously consolidated. However, in the deeper parts of the profile, the energy delivered by the laser was no longer sufficient to intensify the melting, resulting only in minor structural alteration or consolidation, rather than further deepening of the surface profile. The optical surface analysis confirms that laser polishing of vertically oriented samples enables controlled modification of both topographical and structural properties of the surface layer, provided that the laser power and polishing speed are properly optimized. At 10 W, the surface exhibits a regular, periodically repeating relief, where the valley depth and degree of local melting are governed by the energy input, i.e., the polishing speed. At 12 W and lower polishing speeds (*v_f_* = 200 and 400 mm·s^−1^), the higher thermal intensity results in a stochastic surface, with limited presence of periodic reliefs. However, at *v_f_* = 600 mm·s^−1^, a distinct relief with sharper features can still be achieved compared to 10 W. In cases where the objective is to achieve complete surface consolidation and elimination of residual reliefs even at higher polishing speeds, it is necessary to reconsider the polishing parameter setup—particularly the transverse displacement (distance between adjacent laser passes), as this parameter directly affects the overlap of melt pools and thus the resulting surface homogeneity [39,40]. This problem could be solved by design of experiment methodology DOE and the RSM response surface methodology [41].

**Figure 6:**
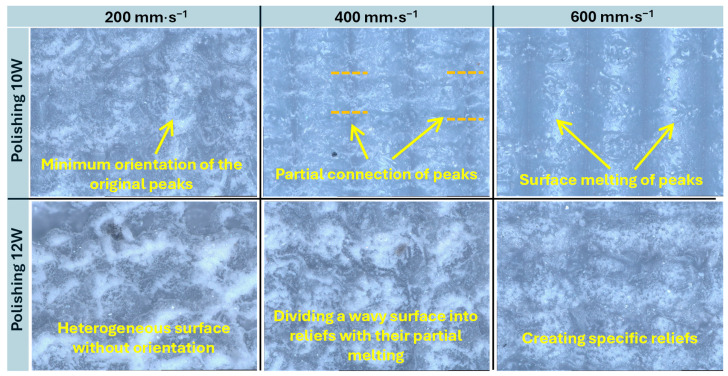
Optical analysis at 1000× magnification of surfaces after laser polishing.

Microscopic analysis at 1000× magnification confirmed the surface topography evolution previously observed in the 3D optical reconstruction. As shown in Figure 6, at 10 W, the original surface peaks were only slightly affected. As the polishing speed decreased to *v_f_* = 400 and 200 mm·s^−1^, progressively stronger interconnection of the peaks occurred due to surface-level melting, which reduced the preferential orientation induced by the printing process. At 12 W and the lowest polishing speed of *v_f_* = 200 mm·s^−1^, complete melting of the surface was observed, with no visible trace of the original print-induced orientation. In contrast, at higher polishing speeds (*v_f_* = 400 and 600 mm·s^−1^), the surface exhibited segmentation into distinct relief structures. At *v_f_* = 400 mm·s^−1^, these reliefs were partially deformed due to higher thermal intensity, whereas at *v_f_* = 600 mm·s^−1^, the shorter exposure time reduced melt depth, resulting in sharper and more clearly defined relief patterns.

### 3.2. Surface Roughness

Table 1 summarizes the surface roughness parameter values for all tested combinations of laser power (*P* = 10 W and 12 W) and polishing speed (*v_f_* = 200, 400, and 600 mm·s^−1^). These data provide a comprehensive insight into the microstructural evolution of surface topography after laser polishing and enable a qualitative assessment of surface phenomena associated with melting, material flow, and subsequent solidification of the polymer matrix. The results clearly demonstrate that the interaction between laser power and polishing speed has a significant impact on the resulting surface topography and the degree of polymer matrix remelting, thereby directly influencing the surface roughness parameters.

**Figure 7:**
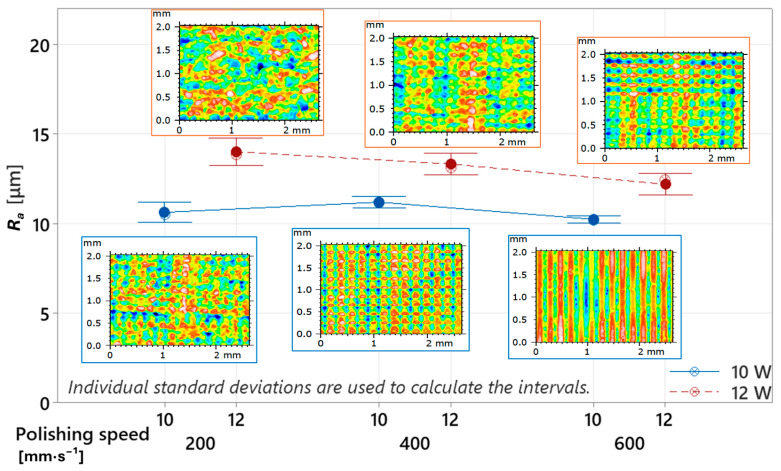
Influence of laser power “*P*” and polishing speed “*v_f_*” on roughness parameter *R_a_*.

As the initial indicator for monitoring surface quality, the arithmetical mean surface roughness (*R_a_*) was selected, as it is the most commonly used parameter for surface roughness evaluation in industrial practice. The graphical trend clearly shows that, under all tested combinations of laser power and polishing speed, higher surface roughness values were consistently recorded at 12 W compared to 10 W (see Figure 7. This difference highlights the strong influence of laser power as the dominant factor determining the melt pool intensity and, consequently, the degree of surface morphology modification. In summary, a higher energy input per unit area results in greater surface irregularities, which is consistent with observations reported in [29]. However, the increased surface roughness at 12 W was also accompanied by a greater data scatter, reflected in the higher standard deviation. This indicates that laser polishing at 12 W resulted in a more heterogeneous surface compared to 10 W. In contrast, lower power (10 W) led to lower variability across all polishing speeds, suggesting a more stable and consistent polishing process. This difference can again be attributed to melt pool intensity [42], as a higher energy concentration at the material surface promotes more aggressive melting of the surface layer, thereby leading to the formation of a rougher and less uniform topography. Comparable surface roughness values of *R_a_* = 12.145 µm and *R_z_* = 64.88 µm were also reported in the study [25], where a picosecond diode laser was used for surface modification. In that study, however, the authors additionally found that the formation of specific surface patterns at this level of roughness could improve hydrophilicity, which further increases the practical relevance of such laser-treated surfaces. Improved hydrophilicity may also be influenced by the specific morphology of the line-shaped valleys formed after the laser pass. Characterization of groove bottoms on PLA processed with a femtosecond laser showed an increase in roughness—i.e., surface irregularities—with increasing energy delivered to the interaction zone [26]. By reducing the delivered energy, it is therefore possible to control the roughness of the groove bottom, which may ultimately affect the functional behavior of the surface.

A comparison of the numerically evaluated average surface roughness values shows *R_a_* = 10.63 ± 0.40 µm, 11.20 ± 0.22 µm, and 10.24 ± 0.14 µm at 10 W, and *R_a_* = 14.01 ± 0.55 µm, 13.34 ± 0.43 µm, and 12.20 ± 0.43 µm at 12 W, for polishing speeds of *v_f_* = 200, 400, and 600 mm·s^−1^, respectively. The results at 12 W clearly indicate a decreasing trend in surface roughness with increasing polishing speed. This behavior is a logical consequence of the reduced laser–material interaction time, which leads to a shallower melt pool and limits excessive remelting of the surface layer [32]. At 10 W, however, a slight increase in roughness was observed at *v_f_* = 400 mm·s^−1^, interrupting the otherwise decreasing trend. This increase in *R_a_* was negligible (0.57 µm) and can therefore be attributed to minor experimental deviations or cumulative process fluctuations occurring during 3D printing or laser polishing—such as localized material inhomogeneities, laser power fluctuations, or small deviations in polishing velocity.

During the 3D printing process, the selected combinations of feed rate and extrusion rate may have caused periodic fluctuations in the printhead motion, which could manifest as micro-vibrations of a certain frequency. Since the specimen was fabricated in the vertical orientation, such micro-oscillations could have induced slight radial deviations between layers, potentially resulting in increased surface roughness due to lateral spreading of the molten filament. However, this effect is considered less probable, as no similar behavior was observed at 12 W, suggesting that its influence on the resulting topography was negligible. Alternatively, it may be assumed that at 10 W and a polishing speed of *v_f_* = 200 mm·s^−1^, the energy input was sufficient to fully melt and partially flow the surface peaks into the valleys, contributing to surface leveling and a reduction in roughness. When the polishing speed increased to *v_f_* = 400 mm·s^−1^, the laser–surface interaction time shortened, reducing the local melt temperature per individual pass. As a result, the molten surface peaks did not have sufficient time to flow and fuse, leading to a rougher and less homogeneous topography. It should be noted that the reference (untreated) samples exhibited an *R_a_* of 9.02 ± 0.21 µm, which is lower than in all laser-polished cases. This indicates that the selected polishing parameters did not smooth the surface but instead increased its roughness. This behavior can be attributed to the micro-relief features observed during optical analysis, generated by the laser beam acting perpendicular to the printed layer orientation. From a physical perspective, the ratio between the laser spot diameter and the transverse displacement (polishing offset) was insufficient, meaning that the adjacent laser tracks did not overlap, leaving untreated intermediate zones and thus increasing microscale topographical irregularity. The resulting surface was therefore rougher and of lower quality. From a practical viewpoint, such parameter configuration may still be advantageous in applications where processing speed is the dominant priority and surface quality is non-critical—particularly in cases focused solely on rapid consolidation of surface layers without functional surface requirements. In contrast, to achieve a smoother and more homogeneous surface, it would be necessary to reduce the transverse displacement, ensuring partial overlap of melt pools, which could improve surface uniformity [24,39]. However, this approach inevitably increases processing time and energy consumption, negatively affecting the overall economic efficiency of the process [30,40]. For this reason, future research on vertically printed specimens must focus on optimizing the balance between surface quality, processing time, and energy input during laser polishing.

**Figure 8:**
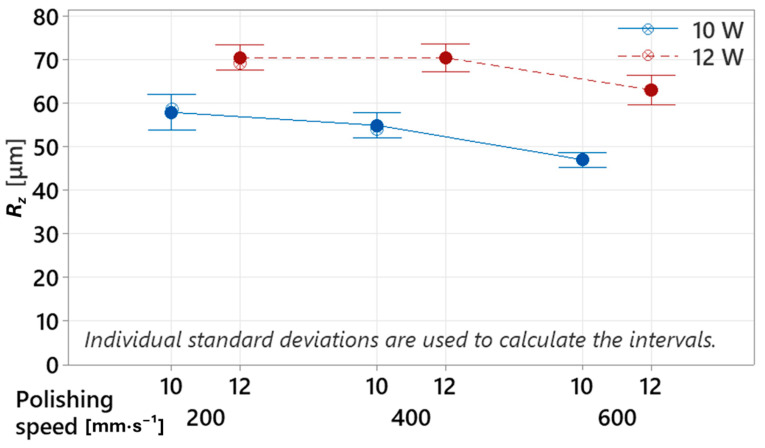
Influence of laser power “*P*” and polishing speed “*v_f_*” on roughness parameter *R_z_*.

Evaluating only the *R_a_* parameter provides a partial and potentially misleading representation of the surface topography, as it expresses merely the arithmetical mean of height deviations along the measured profile. Such a result represents a simplified description of the surface structure, which does not account for the distribution characteristics of the irregularities nor the magnitude of local height extremes. Therefore, to achieve a more comprehensive topographical assessment, the maximum profile height parameter *R_z_* was additionally employed, as it more accurately captures the true amplitude of surface irregularities. The measured *R_z_* values also showed consistently lower values at 10 W (*R_z_* = 57.87 ± 2.92 µm, 54.89 ± 2.05 µm, and 46.99 ± 1.22 µm) compared to 12 W (*R_z_* = 70.46 ± 2.12 µm, 70.40 ± 2.26 µm, and 63.03 ± 2.45 µm) at polishing speeds of *v_f_* = 200, 400, and 600 mm·s^−1^, respectively. However, in contrast to the behavior observed with *R_a_*, the *R_z_* parameter decreased with increasing polishing speed at both 10 W and 12 W (see Figure 8). This contradicts the *R_a_* trend, where a slight increase in roughness was observed at 10 W and *v_f_* = 400 mm·s^−1^. The monotonic decrease in *R_z_* suggests that the previous increase in *R_a_* may have been influenced by isolated local height extremes, which consequently distorted the arithmetic mean. Nevertheless, even the lowest recorded *R_z_* value of 46.99 ± 1.22 µm after laser polishing (10 W, *v_f_* = 600 mm·s^−1^) remained higher than the reference untreated sample (*R_z_* = 44.45 ± 1.64 µm). This confirms that laser polishing resulted in an overall deterioration of surface quality, which could potentially be mitigated by reducing the transverse displacement between laser passes. A smaller spacing would promote more complete surface remelting, thereby reducing the formation of isolated surface reliefs that significantly contribute to increased roughness. Alternatively, laser polishing orientation may also be considered. A theoretical reduction in *R_z_* could be achieved by aligning the laser path parallel to the waviness direction formed during vertical 3D printing. This hypothesis was previously investigated in [43], where parallel polishing resulted in lower surface irregularities compared to perpendicular polishing. However, for vertically oriented samples, such a strategy may risk excessive deepening of existing valleys between printed layers. Therefore, it appears essential to optimize the transverse displacement parameter, ensuring that the laser energy primarily targets the surface peaks, allowing them to melt and flow into adjacent valleys, thereby reducing height differences—and consequently lowering the *R_z_* parameter.

**Figure 9:**
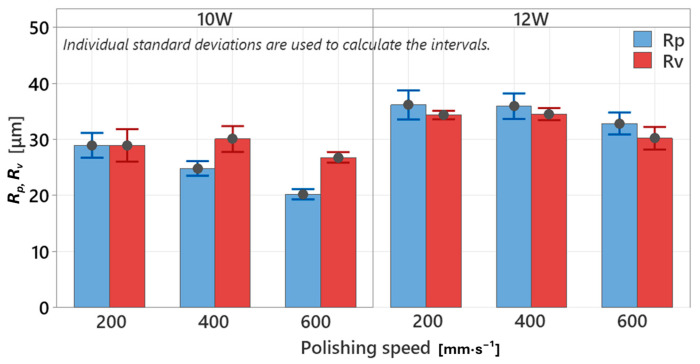
Influence of laser power “*P*” and polishing speed “*v_f_*” on height parameter *R_p_* and *R_v_*.

The analysis of the *R_z_* parameter provided a more precise qualitative assessment of the maximum height difference between the highest peaks and the deepest valleys. However, this height parameter can be further decomposed into two separate components—the mean peak height (*R_p_*) and the mean valley depth (*R_v_*)—measured over the evaluation length. By isolating peak and valley contributions, a more accurate interpretation of surface extremities can be achieved, enabling a deeper understanding of how laser polishing influences surface topography formation. The graphical representation of *R_p_* and *R_v_* dependencies (Figure 9) demonstrates a clear difference in behavior of these parameters under varying laser powers and polishing speeds. At 10 W, the measured peak heights were *R_p_* = 28.95 ± 2.09, 24.82 ± 1.66, and 20.20 ± 0.67 µm, while the valley depths were *R_v_* = 28.93 ± 1.60, 30.07 ± 0.93, and 26.79 ± 0.65 µm, for polishing speeds of *v_f_* = 200, 400, and 600 mm·s^−1^, respectively. This indicates a consistent trend of *R_p_* < *R_v_*, meaning that valleys remain deeper than peaks are tall. This finding aligns with the reference sample, where the peak heights (*R_p_* = 21.41 ± 0.40 µm) were also lower than the valley depths (*R_v_* = 23.05 ± 1.33 µm). It may therefore be concluded that the native surface profile of the reference specimen is predominantly valley-dominated, likely formed by the radial material displacement characteristic of vertically printed FDM layers. After 10 W laser polishing, this profile was partially modified and deepened, yet its fundamental character remained preserved. This suggests that 10 W is capable of thermally fusing the surface layers without significantly altering their functional geometric nature, which may be beneficial when functional surface characteristics must be retained. It is worth noting that the largest difference between *R_p_* and *R_v_* after polishing was observed at the highest polishing speed of *v_f_* = 600 mm·s^−1^ (difference of 6.59 µm). As the polishing speed decreased to *v_f_* = 400 mm·s^−1^, this difference was reduced to 5.25 µm, and at the lowest speed of *v_f_* = 200 mm·s^−1^, the surface approached a near-equilibrium condition, with a difference of only 0.03 µm.

The results indicate that the laser polishing process at 10 W was strongly influenced by the polishing speed, which directly determined the amount of delivered thermal energy, and thus the resulting surface morphology. At the highest polishing speed, the surface morphology remained relatively unchanged, which may be attributed to the higher thermal resistance of the peak regions—whose compact and dense structure (as observed in the optical analysis) was less susceptible to local melting. In contrast, the interlayer regions were more prone to remelting and deepening, due to their porous microstructure, formed during additive layer stacking and radial extrusion effects (as previously described in the optical analysis). As the polishing speed decreased, the difference between peaks and valleys progressively diminished, which can be attributed to the increased melt pool intensity. The higher energy input promoted more uniform melting across both peak and interlayer valley regions, extending into subsurface layers of the material. The longer exposure time enabled partial flow of molten material from the peak areas into the valleys, resulting in surface leveling and a more balanced and homogeneous topographical profile.

This behavior is clearly observed at 12 W. The average peak heights were measured as *R_p_* = 36.13 ± 1.86 µm, 35.91 ± 1.64 µm, and 32.84 ± 1.40 µm, while the valley depths were *R_v_* = 34.33 ± 0.55 µm, 34.50 ± 0.70 µm, and 30.19 ± 1.46 µm, at polishing speeds of *v_f_* = 200, 400, and 600 mm·s^−1^, respectively. In all cases, a consistent *R_p_* > *R_v_* relationship was observed, indicating higher peaks relative to valley depths.

This result can be attributed to the increased thermal intensity of the laser beam at 12 W, which not only melts surface asperities but also penetrates deeper into the valley regions, where the remaining energy is still sufficient to induce subsurface melting. Simultaneously, lateral flow of material from the sidewalls of the surface reliefs (as observed in the optical analysis) contributes to partial filling of the valleys. Such a parameter configuration can therefore be considered potentially advantageous for applications where enhanced homogenization and interlayer consolidation of PLA surfaces is desired—i.e., where the primary objective is not esthetic refinement, but rather structural densification and reinforcement of surface layers. From a physical standpoint, the *R_p_*/*R_v_* ratio reflects the balance between surface melting and solidification dynamics. At 10 W, the profile is dominated by surface-level melting with limited polymer flow, whereas at 12 W, significant subsurface melting and valley backfilling may occur. This shift in thermal-material interaction has a direct impact on functional surface properties, such as heterogeneity [44], roughness [31], and wear resistance [45].

### 3.3. Mechanical Properties

The observed changes in optical and topographical surface characteristics were also reflected in the mechanical response of the material. Tensile tests were conducted on a Testometric X350 universal testing machine at a test speed of 20 mm·min^−1^ under laboratory conditions. The results were compared between untreated reference samples (denoted as ref) and CO_2_ laser-polished samples processed at 10 W and 12 W, with polishing speeds of *v_f_* = 200, 400, and 600 mm·s^−1^ (denoted as: 10 W 200 mm·s^−1^, 10 W 400 mm·s^−1^, 10 W 600 mm·s^−1^, 12 W 200 mm·s^−1^, 12 W 400 mm·s^−1^, 12 W 600 mm·s^−1^), as presented in Table 2. Each laser parameter configuration was applied to five individually 3D-printed specimens, which were subsequently subjected to tensile testing, ensuring statistical reliability of the results.

The reference (unpolished) samples exhibited the following average mechanical properties: Force Peak = 925.10 ± 15.02 N, Stress Peak = 23.13 ± 0.37 MPa, Elongation Peak = 1.89 ± 0.02 mm, and Strain Peak = 2.10 ± 0.03%. These values represent the baseline condition of the material, characterized by interlayer porosity resulting from the FDM process and the distinct surface waviness confirmed through optical analysis. Interestingly, the reference sample (without laser treatment) demonstrated the highest maximum Force Peak (925.10 ± 15.02 N) and Stress Peak (23.13 ± 0.37 MPa) among all tested groups.

At 10 W, all polishing speeds (*v_f_* = 200, 400, and 600 mm·s^−1^) resulted in a slight reduction in Force Peak and Stress Peak (approximately 3–5%), while Elongation Peak and Strain Peak remained nearly unchanged, showing only minimal variations compared to the reference sample. This observation is consistent with the topographical and roughness analyses, which indicated that 10 W primarily induced superficial melting, producing a relatively uniform and slightly smoothed surface without significantly affecting the subsurface structure. It should be noted that no clear trend of improvement or deterioration in tensile performance was observed with varying scanning speeds at this laser power. This indicates that the energy input at 10 W was insufficient to achieve effective bonding between subsurface layers and to consolidate the material structure. Instead, the laser treatment primarily caused superficial segmentation of the printed surface, leading to partial fragmentation. As a result, the thermal effect was too weak to significantly influence the mechanical properties, and the samples exhibited irregular and unpredictable fracture behavior.

At 12 W, the mechanical properties showed a much stronger sensitivity to the laser polishing speed. At the lowest speed (*v_f_* = 200 mm·s^−1^), the sharpest decrease in strength parameters was observed (Force Peak and Stress Peak decreased by approximately 9.3%), correlating with the stochastic surface morphology and surface overheating observed in the optical analysis. Upon increasing the polishing speed to *v_f_* = 400 and 600 mm·s^−1^, the interaction time of the laser beam was reduced, thereby decreasing the thermal input into the material. Although the resulting strength values at these speeds numerically approached the reference data (with Force Peak reductions of 5.3% and only 0.25%, respectively), the differences were small and within the range of the statistical variability of the measurements. Therefore, it is not possible to clearly identify an improvement or recovery of the mechanical properties; however, a reduction in the negative thermal overloading effects observed at the lowest scanning speed can be stated.

These results clearly demonstrate that laser polishing of vertically oriented specimens led to mechanical properties that were comparable to, or slightly inferior to, those of the untreated reference sample. This behavior contrasts with the findings of previous studies [24,32], in which authors reported improvements in both mechanical and topographical properties following optimized laser polishing conditions [29,46]. For example, in study [27], a high-speed Yb-fiber laser was used, and depending on the specific laser processing parameters, the authors achieved a nearly 10% improvement in mechanical properties—such as tensile strength—compared to the untreated sample. At the same scanning speed of *v_f_* = 200 mm·s^−1^, study [28] also reported a 90.4% reduction in *R_a_* together with a 27% increase in tensile strength on horizontally printed samples compared to the untreated specimen when using a fiber laser. However, this improvement was achieved with an almost twice smaller transverse displacement between successive scan tracks 0.04 mm. It is therefore evident that, for vertically built orientations, targeted optimization of the polishing process is essential in order to improve mechanical performance. The optical surface analysis conducted in this study revealed distinct periodic relief patterns, formed by the combination of the waviness produced during printing and the perpendicular laser polishing direction. This repetitive surface segmentation appears to be the primary cause of the deteriorated mechanical response. The formation of such reliefs suggests that the spacing between individual laser polishing tracks was too large, leading not to surface consolidation, but rather to segmentation of the surface topography into smaller regions, thereby creating new potential stress concentrators. Furthermore, it was observed that 10 W resulted in inferior mechanical properties compared to 12 W, which was attributed to the weaker melt pool intensity at the lower power level. Based on these observations, future research should focus on higher laser powers in combination with reduced transverse displacement, in order to achieve sufficiently deep and uniform melting of the surface layer while avoiding the formation of undesired surface reliefs and simultaneously enhancing surface consolidation.

**Figure 10:**
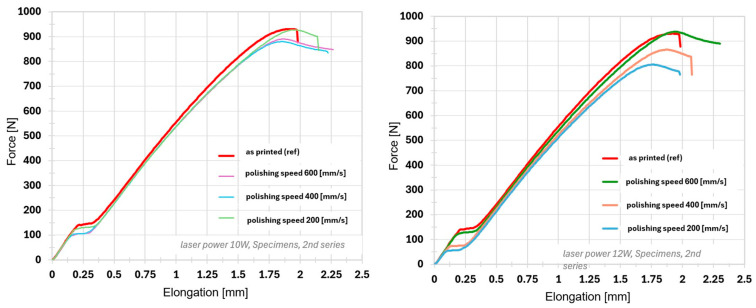
Tensile diagrams showing the course of a tensile test for mechanical properties.

Figure 10 illustrates the tensile stress–strain responses for each combination of laser power and polishing speed in direct comparison with the reference sample. These graphs provide a dynamic representation of the material response under load, complementing the numerical values presented in the table with a visual interpretation of the differences. The inclusion of these diagrams allows a more detailed assessment of the deformation behavior over time and helps confirm the consistency of observed trends across all tested configurations. In addition, such visualization enables identification of the material failure mode, which is further examined in the following section on fracture analysis.

### 3.4. Failure and Facture Characteristics

**Figure 11:**
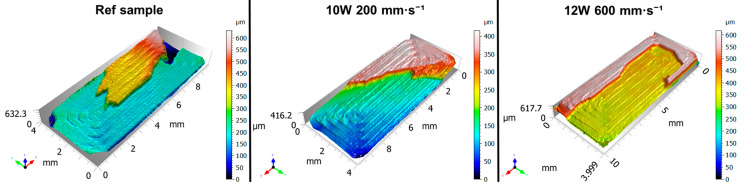
Optical reproduction of the refraction characterized for the reference sample and samples after 10 and 12 W laser polishing.

The fracture morphology of the unpolished sample was chaotic and unpredictable (see Figure 11). The failure occurred across three layers and was positioned near the edge of the specimen. Interestingly, this three-layer fracture initiated precisely at the starting point of the toolpath for a newly deposited layer. This observation suggests that the initial deposition points in the FDM process are the most prone to failure, likely due to the highest probability of defect formation. One possible cause is insufficient bonding between the extruded filament and the previously solidified layer, as the filament temperature may fluctuate slightly at the start of the deposition process [47,48]. Another potential contributing factor is a minor mismatch between the material extrusion rate and the linear feed rate of the nozzle [49]. As previously demonstrated, such inconsistencies can lead to the formation of interfacial voids, which in turn weaken the material [12].Two distinct scenarios may occur in this context:

- The printhead extrudes an excessive amount of material at the starting point due to a temporary delay in XY motion. This can cause over-deposition and local material buildup, which may lead to defects in the subsequent infill process. The excess material prematurely solidifies, and during the following infill passes, the printhead may collide with the hardened material, inducing micro-vibrations or slight displacement of the part, resulting in the accumulation of defects in that region.
- Alternatively, premature XY motion relative to the extrusion rate may occur. In this case, the filament is under-deposited at the starting point, which again leads to poor interlayer bonding and the formation of structurally critical defects.

After the laser polishing process at 10 W, a more favorable fracture morphology was observed compared to the unpolished samples. In this case, the additional remelting of the outer/peripheral walls led to densification of the outer surfaces along the specimen perimeter [23]. Similar positive effects of surface consolidation were also reported in [12,29], where reduced delamination risk was achieved for horizontally printed samples with parallel print orientation relative to the tensile load direction.

At this study, the laser polishing trajectory was perpendicular to the build orientation, resulting in enhanced interlayer bonding. This contributed to a reduction in internal defects and cracks, thereby lowering structural heterogeneity. At polishing speeds of *v_f_* = 400 and 600 mm·s^−1^, the fracture occurred mainly through layer-to-layer separation, without additional delamination of the surrounding material. However, at *v_f_* = 200 mm·s^−1^, the fracture exhibited a shear-like behavior, cutting diagonally through half of a layer. In this case, a defect within the lower infill region was also identified. If this defect had been present prior to the tensile test, it could be considered the initiation site of the fracture, explaining the asymmetric interlayer separation. On the other hand, it may represent a secondary crack formed during failure, indicating the weakest structural zone where fracture propagation began.

Increasing the laser power to 12 W resulted in strong remelting of the peripheral wall and the adjacent subsurface layer. This can be attributed to the higher laser energy input and the associated heat generation at elevated power levels, as previously described in [42]. This was further confirmed by the fracture morphology, where detachment occurred across two adjacent layers. Additionally, delamination within the inner infill of a single layer was observed, while the outer shell walls remained intact, fracturing only at a higher layer position. This indicates a stronger interfacial bonding of the outer shell, which is more resistant to tensile stress, whereas the inner infill region fails earlier, leading to premature detachment or separation.

## 4. Conclusions

This study investigated the surface modification of PLA material using CO_2_ laser polishing, with a specific focus on the vertically oriented build direction, which—to the best of current knowledge—has not yet been systematically explored in this context. The novelty of this work lies in the experimental validation of laser polishing under vertical build conditions. The results demonstrated that adjusting the laser parameters can significantly alter both the surface topography and the mechanical response of the printed parts, clearly confirming that the process efficiency is strongly governed by the manner in which energy is distributed into the surface layer. These findings extend the existing understanding of laser polishing by providing deeper insight into the role of process parameters in controlling melt pool intensity and improving vertically printed components.

The key findings can be summarized as follows:

- The unpolished reference surface exhibited a distinct periodic wavy morphology introduced by the FDM process—characterized by smooth peak regions and porous valleys—confirming the natural anisotropy and interlayer heterogeneity that critically influence mechanical performance.
- 3D surface reconstructions revealed, at both 10 W and 12 W, the formation of periodic relief structures caused by the perpendicular laser polishing direction combined with insufficient hatch overlap. At 10 W, the relief was mildly periodic and partially ordered, whereas at 12 W and lower polishing speeds (*v_f_* = 200–400 mm·s^−1^), the surface became stochastically melted. At *v_f_* = 600 mm·s^−1^, the surface partially stabilized, yet persistent relief features remained due to insufficient overlap of laser passes.
- This insufficient track overlap resulted in increased surface roughness at both energy levels—*R_a_* = 10.24 ± 0.14 µm (10 W) and *R_a_* = 12.20 ± 0.43 µm (12 W) at *v_f_* = 600 mm·s^−1^—in comparison with the reference sample (*R_a_* = 9.02 ± 0.21 µm), confirming the necessity of further optimization of this parameter.
- *R_p_* and *R_v_* analysis showed that at 10 W, valley depth dominated (*R_v_* > *R_p_*), indicating surface-level melting without altering the fundamental FDM topography, whereas at 12 W, *R_p_* exceeded *R_v_* (*R_p_* > *R_v_*) due to intensified subsurface melting and material flow into the valleys.
- The mechanical analysis revealed that the current polishing settings led to a degradation of mechanical performance, attributed to the formation of relief features acting as stress concentrators.
- The fracture analysis confirmed a change in failure mechanism—at 10 W, a more stable layered fracture occurred with improved bonding of the outer shell, while at 12 W, deeper melted zones resulted in fracture initiation within the infill region.

Based on these findings, several key research directions emerge:

- The transverse displacement between laser passes was identified as a critical parameter, as insufficient overlap segmented the original wavy surface into isolated reliefs, increasing roughness and degrading mechanical properties due to stress concentration effects. A systematic optimization of this parameter is essential to enhance both surface quality and mechanical stability.
- A second research opportunity concerns the laser polishing trajectory. In this study, a perpendicular polishing strategy was used relative to the surface waviness from printing. However, a parallel polishing orientation may potentially suppress relief formation, leading to a more compact and uniform surface structure—and should be considered in future studies.

## Figures and Tables

**Table 1:** Parametric comparison of average surface roughness parameters after laser polishing process.

Roughness Parameter	Laser Power *P* [W]						Reference Sample
	10W			12W			
	Laser Polishing Speed *v_f_* [mm·s^−1^]						
	200	400	600	200	400	600	
*R_a_* $\bar{x}$_D_ ± σ_D_ [µm]	10.63 ± 0.40	11.20 ± 0.22	10.24 ± 0.14	14.01 ± 0.55	13.34 ± 0.43	12.20 ± 0.43	9.02 ± 0.21
*R_z_* $\bar{x}$_D_ ± σ_D_ [µm]	57.87 ± 2.92	54.89 ± 2.05	46.99 ± 1.22	70.46 ± 2.12	70.40 ± 2.26	63.03 ± 2.45	44.45 ± 1.64
*R_p_* $\bar{x}$_D_ ± σ_D_ [µm]	28.95 ± 2.09	24.82 ± 1.66	20.20 ± 0.67	36.13 ± 1.86	35.91 ± 1.64	32.84 ± 1.40	21.41 ± 0.40
*R_v_* $\bar{x}$_D_ ± σ_D_ [µm]	28.93 ± 1.60	30.07 ± 0.93	26.79 ± 0.65	34.33 ± 0.55	34.50 ± 0.78	30.19 ± 1.46	23.05 ± 1.33

**Table 2:** Parametric comparison of mechanical properties of samples before and after laser polishing.

Specimen Group	Force Peak [N]	Stress Peak [MPa]	Elongation Peak [mm]	Strain Peak [%]
Ref.	925.10 ± 15.02	23.13 ± 0.37	1.89 ± 0.02	2.10 ± 0.03
10 W 200 mm·s^−1^	896.80 ± 41.28	22.42 ± 1.03	1.92 ± 0.03	2.13 ± 0.03
10 W 400 mm·s^−1^	879.37 ± 13.39	21.98 ± 0.34	1.86 ± 0.01	2.06 ± 0.01
10 W 600 mm·s^−1^	880.26 ± 12.74	22.01 ± 0.31	1.88 ± 0.05	2.09 ± 0.05
12 W 200 mm·s^−1^	838.83 ± 30.49	20.97 ± 0.76	1.81 ± 0.05	2.01 ± 0.05
12 W 400 mm·s^−1^	875.73 ± 13.34	21.89 ± 0.33	1.87 ± 0.01	2.08 ± 0.01
12 W 600 mm·s^−1^	922.79 ± 13.59	23.07 ± 0.34	1.93 ± 0.06	2.14 ± 0.06

## Data Availability

Dataset available on request from the authors.
